# Prevalence and Genotypic Diversity of High-Risk Human Papillomavirus Among Women of Reproductive Age in Kilifi County, Kenya

**DOI:** 10.7759/cureus.83191

**Published:** 2025-04-29

**Authors:** Hellen W Kariuki, Celestine K Nyamari, Peter M Waweru, Patrick M Makazi, Marianne W Mureithi, Wallace Bulimo, Erick Wanjala, Lyle McKinnon, Humphrey N Kariuki, Frank G Onyambu

**Affiliations:** 1 Department of Medical Microbiology and Immunology, University of Nairobi, Nairobi, KEN; 2 Department of Biochemistry, Microbiology and Biotechnology, Kenyatta University, Nairobi, KEN; 3 Department of Molecular Diagnostics and Genomics, Centre for Molecular Biosciences and Genomic, Nairobi, KEN; 4 Department of Medical Physiology, Faculty of Health Sciences, University of Nairobi, Nairobi, KEN; 5 Department of Neglected Tropical Diseases (NTDs), County Government of Kilifi, Kilifi, KEN; 6 Department of Medical Microbiology and Immunology, Faculty of Health Sciences, University of Nairobi, Nairobi, KEN; 7 Department of Epidemiology, Statistics and Informatics, Kenya Medical Research Institute (KEMRI), Nairobi, KEN; 8 Department of Reproductive Health, Kilifi County Hospital, Kilifi, KEN; 9 Department of Reproductive Health, Pwani Gyno Centre, Mombasa, KEN; 10 Department of Medical Microbiology and Infectious Diseases, University of Manitoba, Winnipeg, CAN; 11 Mucosal Immunology Laboratory, Centre for the AIDS Programme of Research in South Africa (CAPRISA), Durban, ZAF; 12 Department of Medical Laboratory Sciences, Meru University of Science and Technology, Meru, KEN

**Keywords:** hrhpv, hrhpv 16, hrhpv 18, hrhpv 45, kenya, kilifi, magarini, rabai, women

## Abstract

Background

Human papillomavirus (HPV) is the most common sexually transmitted infection and the primary cause of cervical cancer, a leading cause of cancer-related deaths among women in Kenya. Although many HPV infections resolve on their own, some high-risk types may persist and gradually develop into cervical cancer over several years, providing opportunities for early detection and intervention. However, in low-resource settings like Kilifi County, HPV testing is limited, and alternative screening methods like visual inspection with acetic acid (VIA) are commonly used despite their limitations.

Objective

This study aimed to assess the prevalence and genotype distribution of high-risk HPV (HR-HPV) among women of reproductive age in Kilifi County, Kenya, to inform targeted public health interventions.

Methodology

This study was nested within a more extensive cross-sectional study on female genital schistosomiasis and human immunodeficiency virus (HIV). We focus on a stratified sample of 320 women aged 15-50 from Rabai and Magarini sub-counties, Kilifi, Kenya, identified as *Schistosoma haematobium* hotspots. Participants provided informed consent, and pregnant women were excluded. Clinical data was collected and sociodemographic data collected via questionnaires, while high vaginal and cervical swabs were self-collected for HPV testing, screening for 24 HR-HPV genotypes.

Results

Data from 261 women were analyzed. The overall HR-HPV prevalence was 48.7%, with the Magarini sub-county showing a higher prevalence (31.4%) compared to Rabai (17.2%). The most prevalent HPV genotypes were HPV 18 (25.3%), HPV 45 (22.6%), and HPV 16 (12.6%). Co-infections were common, particularly with HPV 18 and 45. HPV 16 was more prevalent in the Rabai subcounty, while HPV 18 and 45 were more common in the Magarini subcounty. Significant associations were found between sexual partnership type, leukocyte levels, and HPV positivity.

Conclusion

Kilifi County exhibits a high prevalence of HR-HPV, with genotype variations across sub-counties, suggesting differences in risk factors and access to preventive measures. Self-sampling and community-based screening effectively increased participation and diversity in the study population, highlighting the need for targeted, age-specific screening programs and comprehensive HPV genotyping to enhance cervical cancer prevention strategies in the region.

## Introduction

Human papillomavirus (HPV), the most prevalent sexually transmitted infection (STI) worldwide, is the primary cause of cervical cancer, the fourth most prevalent cancer globally [1]. In 2020, 604,127 new cervical cancer cases and 341,831 deaths were reported, with sub-Saharan Africa accounting for the second-highest burden [2]. In Kenya, cervical cancer accounted for 5,845 incidences and 3,591 deaths in 2022 [3]. HPV-related cancers are the leading cause of mortality among the cancers reported in Kenya, with cervical cancer being the second most common female cancer in women aged 15-44 years [4].

HPVs are classified broadly into low-risk HPVs (LR-HPVs) and high-risk HPVs (HR-HPVs). The LR-HPVs cause benign cutaneous and anogenital warts while HR-HPVs cause oropharyngeal, anal, cervical, vaginal, vulvar, and penile cancers [5]. HR-HPV types 16 and 18 are responsible for 71% of all cervical cancer cases worldwide [6]. HPV 16, 18, and 45 are attributed to 94% of cervical adenocarcinomas [7]. The other oncogenic types are HPV 31, 33, 35, 39, 51, 52, 56, 58, 59, 68, 73, and 82, which account for an additional 20% of cervical cancer cases [8].

Although HPV is highly transmissible with a lifetime risk of 50-70% in sexually active women, the majority of infections clear up spontaneously in two to three years due to cell-mediated immunity. However, some HR-HPV infections persist, leading to pre-cancerous lesions [9]. Progression of HPV-infected epithelial cells to invasive cancer is a long-term process (usually 10-12 years) traditionally associated with the accumulation of DNA alterations in host cell genes [10]. HPV pathogenesis involves the overexpression of viral oncoproteins that affect cell proliferation, cell cycle, and apoptosis, leading to cervical carcinogenesis [11,12]. The premalignant changes, known as cervical intraepithelial neoplasia (CIN), are graded from CIN1 to CIN3 based on the severity of cervical epithelial abnormalities. If left untreated, these changes can progress to invasive cervical cancer [10]. Other factors contributing to the development of cervical cancer include early onset of sexual debut, co-infection with other STIs, multiparity, contraceptive use, and tobacco use [13].

Co-infection with HR-HPV infections is frequently detected among women with abnormal cervical cytology or histology, with prevalence rates ranging from 20% to over 50% [14]. Multiple infections have been known to increase the risk of CIN3 or higher due to additive or synergistic effects, leading to higher rates of disease progression or recurrence post-treatment [15]. One study reported that coinfection with multiple HPV types, especially from the same clade, increases the risk of developing CIN2+. However, for HPV16 and HPV18, additional coinfections did not significantly alter the risk of CIN2 [16]. Several factors are suggested to influence these coinfections, including the type of population studied, geographic clustering, age, type of specimen (cytology vs. histology), recent sexual history, HIV infection, and the sensitivity of the genotyping system used [17].

The prolonged duration for the development of cervical cancer provides a window for the early detection and treatment of precancerous lesions. Cervical cancer screening programs have proved effective in reducing the burden of this disease in most high-income countries [18,19]. However, several African countries, Kenya included, struggle to maintain widespread screening programs. In Kenya, screening methods include visual inspection with acetic acid or visual inspection using lugol’s iodine (VIA/VILI), pap smear test, and HPV deoxyribonucleic acid (DNA) testing, the latter being recommended as the primary screening method by both the World Health Organization's Global Cervical Cancer Elimination Strategy and the Kenyan cancer screening guidelines [20,21]. Although in the majority of areas in Kenya, including Kilifi County, HPV testing is not widely available in the public healthcare system, and where it is available, the majority of women are unable to afford to pay for the test.

In Kilifi County, the common method used for screening is VIA/VILI due to the limited infrastructure and inadequate resources. However, this test is usually associated with a high false-positive rate [22,23]. Pap smears available in some hospitals are usually associated with challenges including the subjective interpretation of cytological slides, which is prone to inter-observer variability, the risk of missing the lesion area during sampling, inadequate preservation of cells, low sensitivity in detecting pre-invasive and invasive glandular lesions, and the reporting of false negatives [24]. In contrast, the HPV polymerase chain reaction (PCR) test has a high negative predictive value and high sensitivity for detecting precancerous lesions, making it effective when combined with cytology for early detection [25]. However, this test is not widely available in low-resource settings. As a result, many women with cervical cancer may have never been screened for HR-HPV, with interventions often occurring only after the appearance of abnormal symptoms.

This study is part of a larger investigation on the effect of female genital schistosomiasis and HIV infection, specifically examining the effect of *Schistosoma haematobium* on α4β7 expression among Kenyan women. We conducted a cross-sectional study among women of reproductive age in Kilifi County to assess the prevalence and genotype distribution of HR-HPV in this population.

## Materials and methods

Study area and study setting

The HPV study is nested in a cross-sectional study that investigated female genital schistosomiasis and HIV infection in women of reproductive age in Kilifi County, which recruited women of reproductive age in Rabai and Magarini sub-counties in Kilifi County. The inclusion criteria included freely consenting to participate, being a woman aged 15-50 years, and being a resident of the study area. Pregnant women and those who had not abstained from sexual intercourse for 48 hours before sample collection were excluded from the study.

The study was conducted in Kilifi County, specifically in the Rabai and Magarini sub-counties, which are hotspots for *S. haematobium *in the region, from September 2022 to November 2022. In Rabai sub-county, the study was undertaken in Ruruma Ward in two sub-locations: Jimba sub-location and Mleji sub-location. In the Magarini sub-county, the study was undertaken in three wards: Magarini, Garashi, and Sabaki wards. In Magarini Ward, the following sub-locations were sampled: Mwangatini sub-location and Burangi sub-location. In Garashi Ward, the following sub-locations were sampled: Bore Singwanya sub-location, Masindeni sub-location, and Mikuyuni sub-location, and Sabaki Ward, Sabaki sub-location along the river Sabaki.

Study population and sampling technique

Rabai and Magarini sub-counties were identified as *S. haematobium* hot spots based on their topography and high prevalence of urogenital schistosomiasis in school-going children by the Neglected Tropical Disease in Kilifi County. The number of women to be sampled from the specific sub-county was calculated from the total number of women of reproductive age divided by the expected sample size per county (150) to get the sampling interval. In each village, the number of women was divided by the sampling interval to obtain a representative number of women per village. Women were randomly selected from each household, with one woman representing a specific household. If more than one woman was eligible, one was randomly selected to represent that household. Women aged 15 to 50 years who had abstained from sexual intercourse for at least 48 hours were recruited for the study. Exclusion criteria included pregnant women, virgins, and individuals who had engaged in sexual activity within the 48 hours before sample collection. From the consented women, both high vaginal swabs (HVS) and vaginal swabs were collected by self-swabbing.

Questionnaire administration

A questionnaire was used to collect the sociodemographic data that included the economic activities, water activities, education level, and other potential risk factors that predispose women to *S. haematobium*, HIV, and HPV infection.

Data collection and sample collection

HVS Collection

The participant held a six-inch vaginal swab and inserted the swab vaginally until their fingers touched the labia minora. The swab was moved in a circular motion against the vaginal walls for a minimum of 15 repetitions. The collected swab was placed back in the swab holder. For the cervical swab/HVS, the participant inserted a six-inch flocked swab vaginally until they met noticeable resistance. Then performed the swab rotation that is moving the swab in a circular motion against the vaginal walls for a minimum of 15 repetitions. The shaft was broken and the swab was placed in a sterile screw cup microtubes. The samples were labelled with unique identifiers, put in a cooler box to preserve the DNA, ribonucleic acid (RNA), and microbiota, and stored at -20°C at the Kilifi County Hospital for further processing.

HPV Genotyping

Cervical samples collected using flocked swabs were resuspended in 1 mL of phosphate-buffered saline (PBS) to release cellular material. DNA was extracted using the GeneProof Pathogen-Free DNA Isolation Kit (GeneProof, Czech Republic), following the manufacturer’s instructions. Briefly, 200 µL of the resulting cell suspension was used as the starting material. Following lysis, DNA was bound to a silica membrane using spin columns, washed to remove impurities, and eluted in 50 µL of elution buffer.

HR-HPV detection and genotyping were performed using the GeneProof High-Risk HPV PCR Kit (GeneProof, Czech Republic), which targets the E1/E2 regions of the HPV genome and identifies 24 HR-HPV genotypes: 16, 18, 26, 30, 31, 33, 34, 35, 39, 45, 51, 52, 53, 56, 58, 59, 66, 67, 68, 69, 70, 73, 82, and 97. The assay differentiates HPV types 16, 18, and 45 through real-time PCR. PCR reactions were prepared using 15 µL of PCR master mix and 5 µL of purified DNA, for a final reaction volume of 20 µL. The amplification protocol consisted of the following thermal cycling conditions: initial hold at 42°C for 15 minutes (one cycle), second hold at 95°C for 10 minutes (one cycle), followed by 45 cycles of 95°C for five seconds, 60°C for 40 seconds, and 72°C for 20 seconds. A final hold at 10°C for one minute was included. Fluorescent detection was performed using the following channels: FAM for HR-HPV, HEX for the internal control (GAPDH), CY5 for HPV 16, Texas Red for HPV 18, and Quasar 705 for HPV 45. PCR was performed using the Bio-Rad CFX96 Real-Time PCR System (Bio-Rad Laboratories, USA). All laboratory procedures were conducted at the CMB Genomics Laboratory in Nairobi, Kenya.

Ethical approval

Ethical clearance was sought from the Kenyatta National Hospital-University of Nairobi Ethical Research Committee (P334/05/2018) and subsequent approval reference by the National Commission for Science, Technology and Innovation, research number NACOSTI/P/22/1769 and Kilifi County Health Research Committee. Data was coded using a unique identifier linked to the names of the participants and stored confidentially by the lead investigator.

Statistical analysis

All collected data were entered and stored in Microsoft Excel (Microsoft® Corp., Redmond, WA, USA). The raw data from the questionnaires and HPV results were cleaned and reviewed for completeness and consistency. Unmatched results and incomplete questionnaires with missing data were excluded from the analysis. Socio-demographic and behavioural data were coded. Data analysis was performed using IBM SPSS Statistics for Windows, Version 26 (Released 2018; IBM Corp., Armonk, New York, United States). Sociodemographic characteristics, such as age, marital status, education level, and occupation, were summarized using frequency tables. The prevalence of HR-HPV was determined, and percentiles were used to describe the distribution of HPV positivity within our study population. The distribution of specific HPV genotypes was also determined, with results expressed as percentages of the total HPV-positive cases. Data is presented in graphs to represent key findings visually. The association between HPV status, sociodemographic characteristics, and clinical characteristics was first examined using the chi-square test. Logistic regression was then performed to study the relationship between HPV status and multiple independent variables to assess the risk factors for HPV infection. The strength of these associations was quantified using odds ratios (ORs) and 95% confidence intervals (CIs). A p-value of <0.05 was considered statistically significant.

## Results

A total of 320 genital swabs were collected from consenting women of reproductive age in Rabai and Magarini sub-counties in Kilifi County. Of the 320 samples analysed, 291 were included in the study, with only 261 participants completing the questionnaire. Therefore, the analysis focused on data from these 261 participants who had complete data sets to determine the prevalence and risk factors associated with HR-HPV infection in the two sub-counties of Kilifi County. Among the 261 participants, 111 were from the Rabai sub-county, specifically from the Jimba and Mleji sub-locations in the Ruruma ward. The remaining 150 participants were from the Magarini sub-county, which included three wards: Magarini, Garashi, and Sabaki. The majority of the participants were aged between 30 and 39 years (n=103, 39.5%) followed by 40 and 49 years (n=73, 28%), 20 and 29 years (n=72, 27.6%), 15 and 19 years (n=7, 27%), and 50 and 59 (n=6, 3%). Out of the 261 participants, 240 (92%) were married, 145 (55.6%) were under customary law, 151 (57.9%) had a primary level of education, and 161 (61.7%) were farmers. The social demographic characteristics of the included participants are described in Table 1.

**Table 1:** Characteristics of the study participants

Characteristics	N	%	Rabai, n (%)	Magarini, n (%)
Age group (years)				
15-19	7	2.7	1 (0.4)	6 (2.3)
20-29	72	27.6	23 (8.8)	49 (18.8)
30-39	103	39.5	45 (17.24)	58 (22.22)
40-49	73	28	37 (14.2)	36 (13.8)
50-59	6	2.3	5 (1.9)	1 (0.4)
Marital status				
Married/living as married	240	92	101 (38.7)	139 (53.3)
Separated/divorced	9	3.4	4 (1.5)	5 (1.9)
Never	6	2.3	3 (1.14)	3 (1.14)
Widowed	6	2.3	3 (1.14)	3 (1.14)
Type of marriage				
Customary	145	55.6	23 (12.64)	112 (49.91)
Civil	7	2.7	4 (1.53)	3 (1.14)
Consensual	65	24.9	49 (18.8)	16 (6.1)
Never	44	16.9	25 (9.6)	19 (7.3)
Education				
None	92	35.2	38 (14.6)	54 (20.7)
Primary	151	57.9	64 (24.52)	87 (33.33)
Secondary	18	6.9	9 (3.44)	9 (3.44)
Occupation				
Casual labourer	10	3.8	8 (3.1)	2 (0.8)
Business	45	17.2	13 (5.0)	32 (12.26)
Farmer	161	61.7	47 (18.0)	114 (43.7)
Housewife	45	17.2	43 (16.5)	2 (0.8)

HPV prevalence in Kilifi County

**Figure 1:**
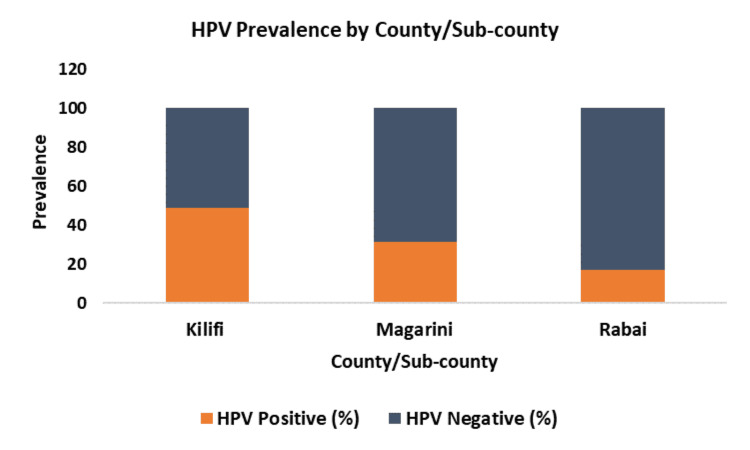
HPV prevalence within Kilifi County, Magarini, and Rabai sub-counties HPV: human papillomavirus

The overall prevalence of HR-HPV in Kilifi County was 48.7% (127 out of 261 participants). Magarini sub-county had a higher prevalence of 31.4% (82 out of 261 participants) when compared to Rabai sub-county, where the prevalence was 17.2% (45 out of 261 participants) (Figure 1).

HR-HPV prevalence within wards in Rabai sub-county

**Figure 2:**
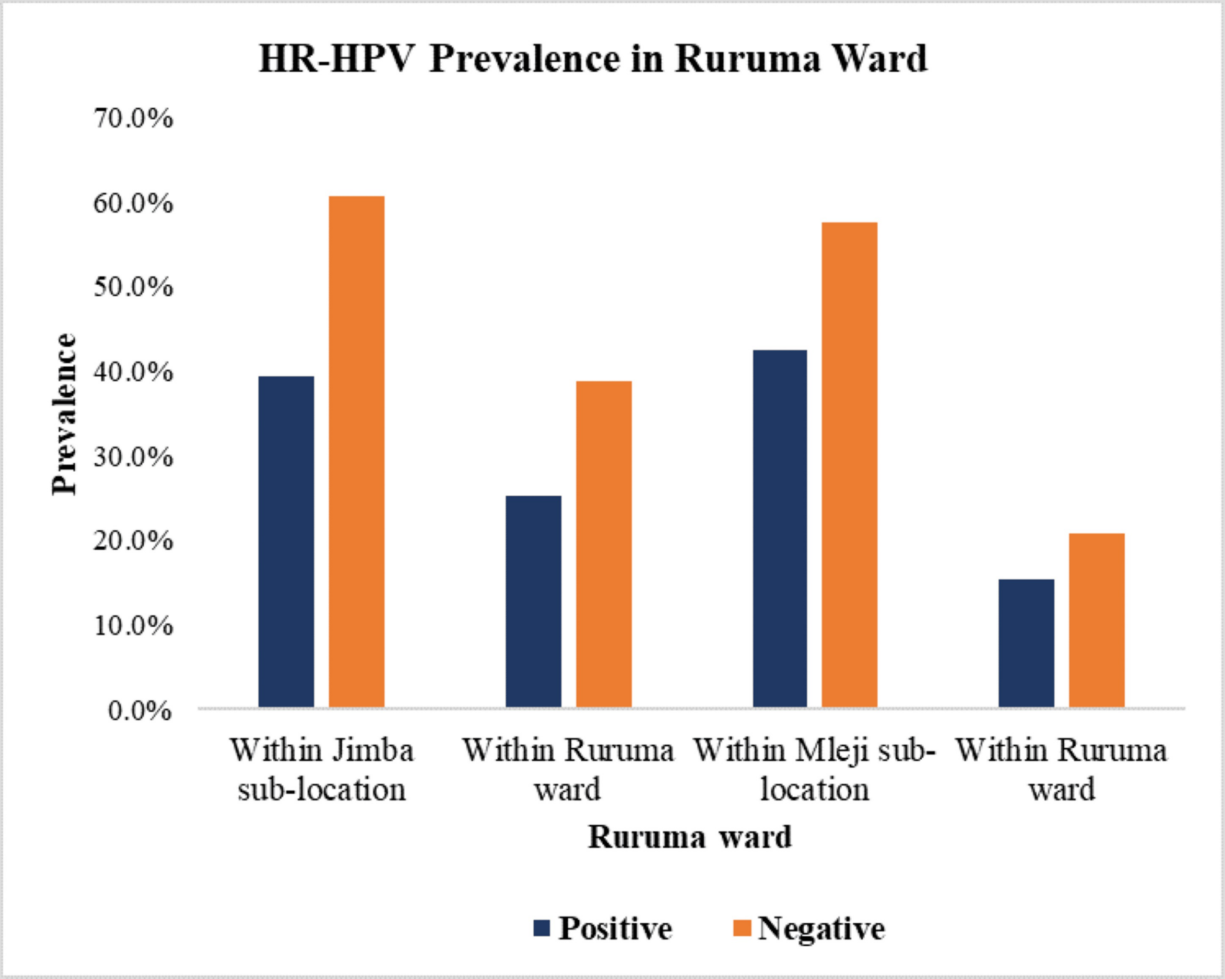
Prevalence of HR-HPV in Ruruma ward in Jimba and Mleji sub-locations within the sub-locations and within Ruruma ward, respectively HR-HPV: high-risk human papillomavirus

**Figure 3:**
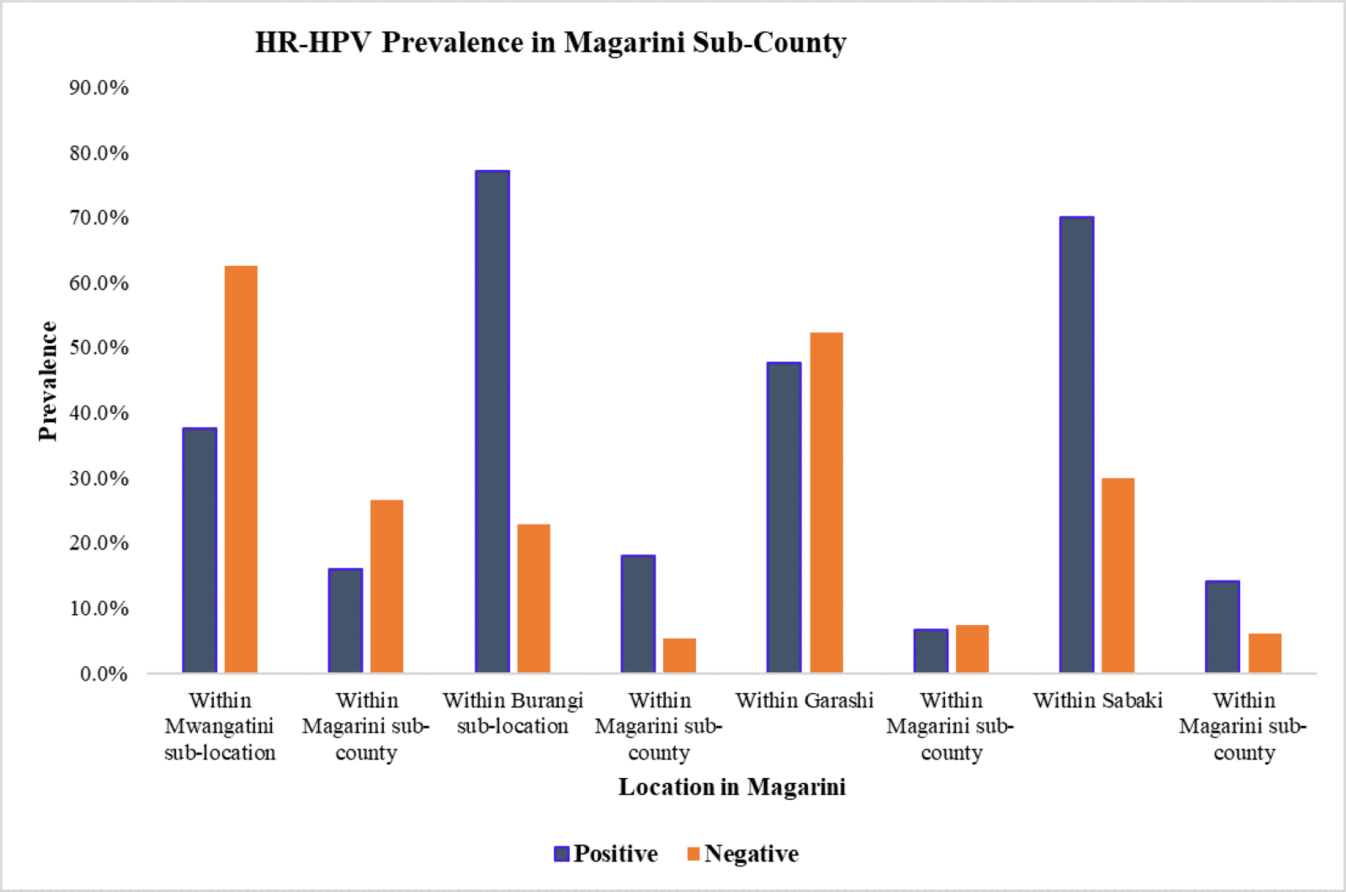
Prevalence of HR-HPV in Mwangatini and Burangi sublocation in Magarini ward, Garashi and Sabaki wards and within the Magarini sub-county, respectively HR-HPV: high-risk human papillomavirus

In Jimba sub-location, Ruruma ward, Rabai sub-county, a total of 111 participants were tested, with 28 (25.2%) testing positive for HR-HPV. In Mleji, out of the 40 participants, 17 (15.3%, 17/111) were positive for HR-HPV (Figure 2). In the Magarini sub-county, 150 participants were tested for HR-HPV. In Magarini ward, Mwangatini sub-location had 24 out of 64 participants (16%) testing positive for HR-HPV, while Burangi sub-location had 27 out of 35 participants (18%) testing positive for HR-HPV. Garashi ward had 10 out of 21 participants (6.7%) testing positive for HR-HPV, while Sabaki sub-location had 21 out of 30 (14%) testing positive for HR-HPV (Figure 3).

HPV status by age groups

**Figure 4:**
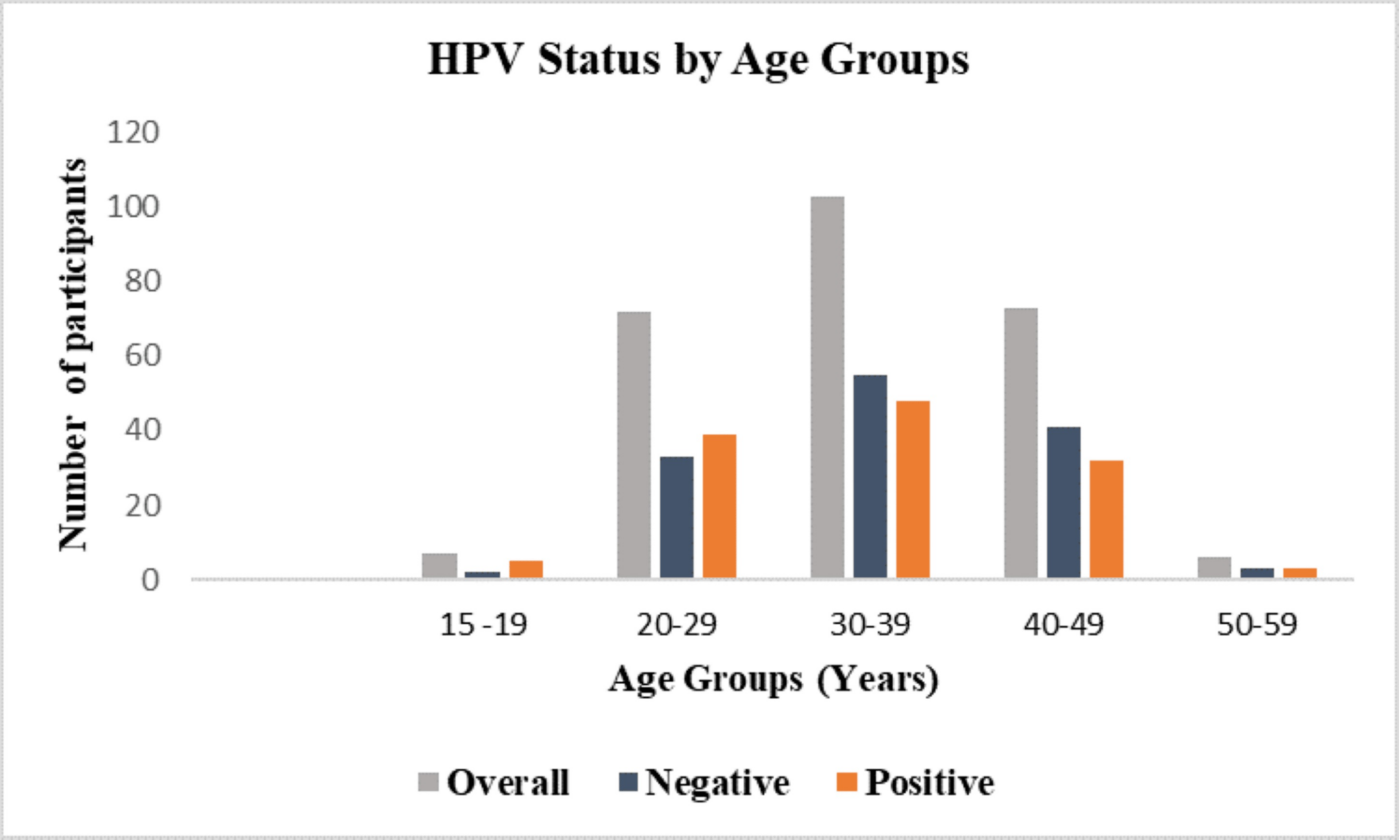
HPV distribution within the different age groups HPV: human papillomavirus

A total of seven participants aged 15-19 were enrolled in the study, with five testing positive for HPV. Among participants aged 20-29, 72 were enrolled, and 39 were HPV positive. The highest number of participants (n=103) was within the 30-39 age group, with 48 testing positive for HPV. In the 40-49 age group, 73 participants were enrolled, with 32 testing positive. The 50-59 age group had the lowest participants, with six enrolled, and three of these were HPV positive (Figure 4).

Prevalence of HPV strains in Kilifi County

**Figure 5:**
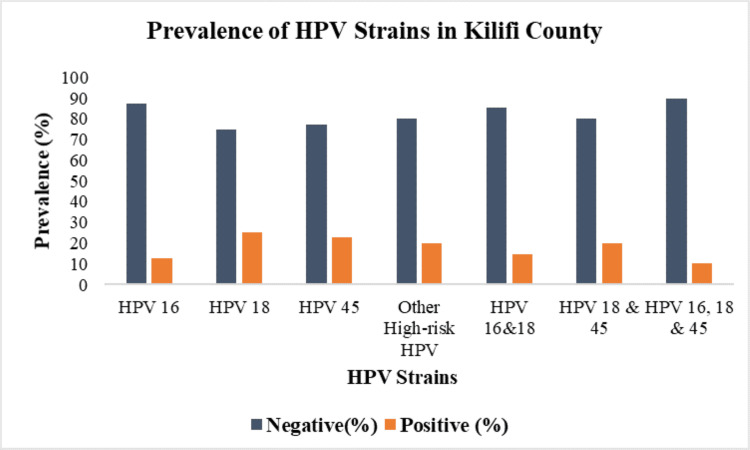
Prevalence of HPV strains in Kilifi County HPV: human papillomavirus

Three HPV strains were identified: HPV 16, 18, and 45. Additionally, there were other undifferentiated HR-HPV strains, including HPV 26, 30, 31, 33, 34, 35, 39, 51, 52, 53, 56, 58, 59, 66, 67, 68, 69, 70, 73, 82, and 97. These strains were not differentiated in the analysis. HPV 18 had the highest prevalence at 25.3% (n=66), followed by HPV 45 at 22.6% (n=59), and other HR-HPV types at 19.9% (n=52). HPV 16 had the lowest prevalence at 12.6% (n=33). We observed co-infections with multiple HPV strains, with 19.9% (n=52) of participants having co-infection with HPV 18 and 45, 14.6% (n=38) with HPV 16 and 18, and 10.3% (n=27) having co-infection with HPV 16, 18, and 45. No co-infection was found with HPV 16 and 45 (Figure 5).

Distribution of HPV strains in Rabai and Magarini sub-counties

**Figure 6:**
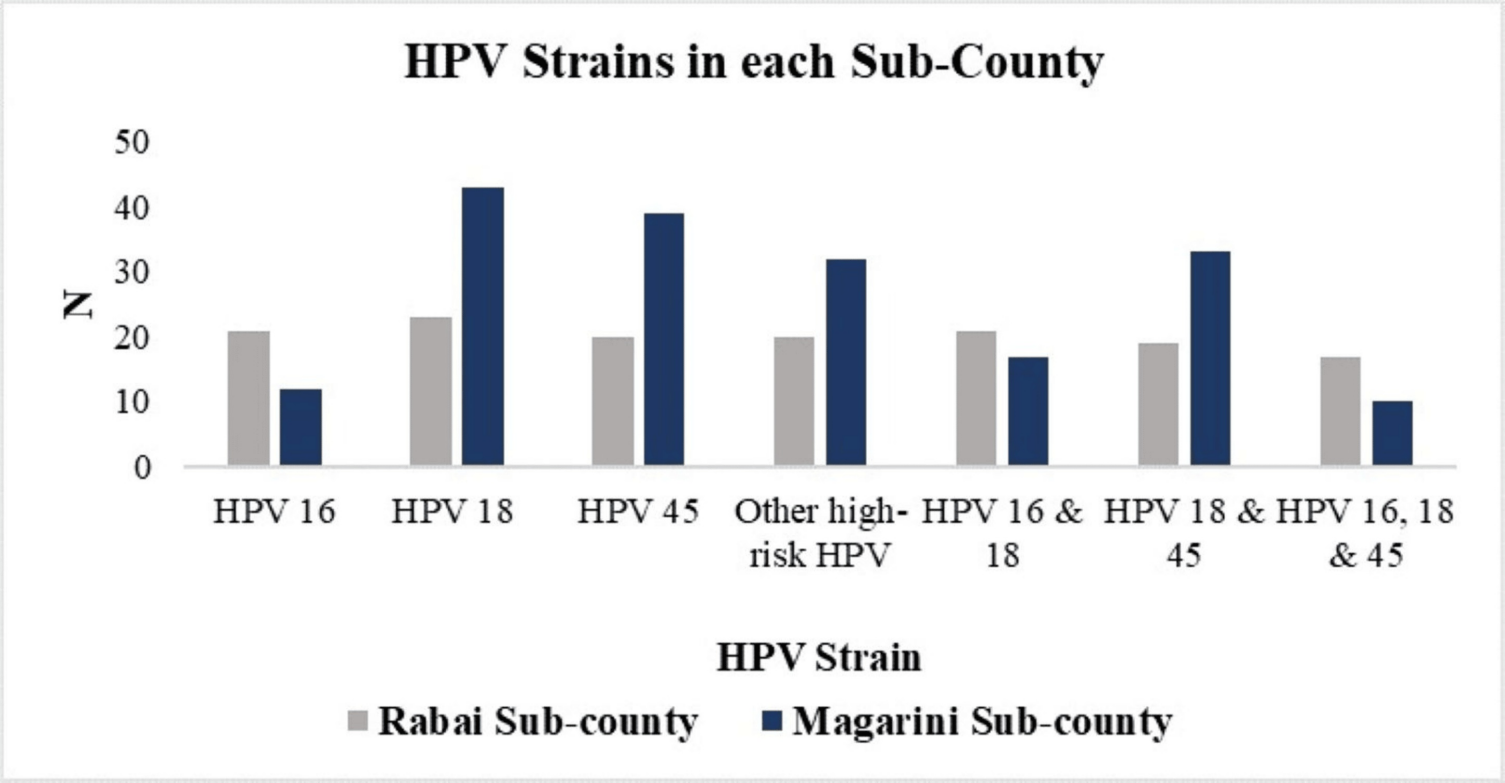
Distribution of HPV strains in Rabai and Magarini sub-counties in Kilifi County HPV: human papillomavirus

HPV 16 was more prevalent in Rabai (n=21) compared to Magarini (n=12). Conversely, HPV 18 was more prevalent in Magarini (n=43) than in Rabai (n=23). Similarly, HPV 45 had a higher prevalence in Magarini (n=39) compared to Rabai (n=20). Magarini also had higher numbers of other HR-HPV types (n=32) than Rabai (n=20). A total of 38 women were co-infected with HPV 16 and 18 (21 in Rabai and 17 in Magarini), and 52 women had co-infection with HPV 18 and HPV 45 (19 in Rabai and 33 in Magarini). A total of 27 women had co-infection with HPV 16, 18, and 45 (17 in Rabai and 10 in Magarini). Co-infection rates were higher in Magarini than in Rabai, with the highest being co-infection with HPV 18 and 45 (Figure 6).

Association between HPV status and participant demographic and clinical characteristics

The association between HPV status and sociodemographic characteristics and behaviour is summarized in Table 2. Chi-square test results revealed a significant association between the type of sexual partnership (monogamous vs. polygamous) and HPV status (p=0.04) with an OR of 0.6 (95% CI 0.3-1.0). There was a significant association between leukocytes and HPV status (p=0.005), suggesting an association between higher leukocyte levels and HPV positivity.

**Table 2:** HPV status by sociodemographic characteristics and behaviour HPV: human papillomavirus; IUD: intrauterine device

Characteristics	Overall (N=261)	Positive (N=127)	Negative (N=134)	P-value
Condom use				0.5
Yes	3 (1.1%)	2 (1.6%)	1 (0.7%)	
No	258 (98.9%)	125 (98.4%)	133 (99.3%)	
Sexual partner				0.04
Monogamous	190 (72.8%)	85 (66.9%)	105 (78.4%)	
Polygamous	71 (27.2%)	42 (33.1%)	29 (21.6%)	
Contraceptive use				0.57
Yes	166 (63.6%)	83 (65.4%)	83 (61.9%)	
No	95 (36.4%)	44 (34.6%)	51 (38.1%)	
Age of sexual debut				0.17
15-19	157 (60.2%)	84 (62.7%)	73 (57.5%)	
20-29	65 (24.9%)	36 (26.9%)	29 (22.8%)	
30-39	2 (0.8%)	1 (0.7%)	1 (0.8%)	
<15	11 (4.2%)	2 (1.5%)	9 (7.1%)	
Unknown	26 (10.0%)	11 (8.2%)	15 (11.8%)	
Type of contraceptive				0.201
Pills	24 (9.2%)	11 (8.2%)	13 (10.2%)	
IUD	35 (13.4%)	20 (14.9%)	15 (11.8%)	
Implant	66 (25.3%)	29 (21.6%)	37 (29.1%)	
Diaphragm/foam/jelly	16 (6.1%)	13 (9.7%)	3 (2.4%)	
Condom	4 (1.5%)	1 (0.7%)	3 (2.4%)	
Safe period	14 (5.4%)	6 (4.5%)	8 (6.3%)	
Sterilisation	3 (1.1%)	2 (1.5%)	1 (0.8%)	
None	99 (37.9%)	52 (38.8%)	47 (37%)	
Education level				0.533
None	92 (35.2%)	43 (33.9%)	49 (36.6%)	
Primary	151 (57.9%)	73 (57.5%)	78 (58.2%)	
Secondary	18 (6.9%)	11 (8.7%)	7 (5.2%)	
Marital status				0.556
Married/living as married	240 (92.0%)	114 (89.8%)	126 (94.0%)	
Separated/divorced	9 (3.4%)	6 (4.7%)	3 (2.2%)	
Never married	3 (1.1%)	3 (2.4%)	6 (4.5%)	
Widowed	4 (1.5%)	2 (1.6%)	6 (4.5%)	
Nitrites				0.744
Positive	9 (3.4%)	5 (3.9%)	4 (3.0%)	
Negative	252 (96.6%)	122 (96.1%)	130 (97%)	
Leukocytes				0.005
++	9 (3.4%)	4 (3.1%)	5 (3.7%)	
+++	22 (8.4%)	18 (14.2%)	4 (3.0%)	
Negative	230 (88.1%)	105 (82.7%)	125 (93.3%)	

Factors associated with HPV infection

Logistic regression analysis of HPV status and multiple variables in Table 3 showed that diaphragm/foam/jelly was significantly associated with a higher likelihood of being HPV positive (p=0.042) with an OR of 3.917 (95% CI 1.051-14.6). The wide CI suggests some uncertainty about the exact magnitude of this effect. There was also a significant association between leukocytes and HPV status (p=0.013). The relationship between high leukocyte level (+++) and HPV status was statistically significant (p=0.003) with an OR of 0.187 (95% CI 0.061-0.561).

**Table 3:** Logistics regression analysis of variables associated with HPV infection HPV: human papillomavirus; IUD: intrauterine device

Characteristics	Overall (N=261)	Negative (N=134)	Positive (N=127)	OR (95% CI)	P-value
Age groups					0.546
15-19	7 (2.7%)	2 (1.5%)	5 (3.9%)	0.4 (0.04-4.0)	0.433
20-29	72 (27.6%)	33 (24.6%)	39 (30.7%)	0.85 (0.16-4.48)	0.844
30-39	103 (39.5%)	55 (41.0%)	48 (37.85)	1.146 (0.221-5.945)	0.871
40-49	73 (28.0%)	41 (30.6%)	32 (25.2%)	1.281 (0.242-6.777)	0.771
50-59	6 (2.3%)	3 (2.2%)	3 (2.3%)	Ref	
Marital status					0.574
Married/living as married	240 (92.0%)	114 (89.8%)	126 (94.0%)	2.21 (0.397-12.3)	0.365
Separated/divorced	9 (3.4%)	6 (4.7%)	3 (2.2%)	1.0 (0.112-8.95)	1
Never married	3 (1.1%)	3 (2.4%)	6 (4.5%)	2.0 (0.194-20.6)	0.56
Widowed	4 (1.5%)	2 (1.6%)	6 (4.5%)	Ref	
Education level					0.539
None	92 (35.2%)	43 (33.9%)	49 (36.6%)	1.791 (0.638-5.028)	0.269
Primary	151 (57.9%)	73 (57.5%)	78 (58.2%)	1.679 (0.618-4.564)	0.31
Secondary	18 (6.9%)	11 (8.7%)	7 (5.2%)	Ref	
Type of contraceptive					0.27
Pills	24 (9.2%)	11 (8.2%)	13 (10.2%)	0.77 (0.313-1.871)	0.557
IUD	35 (13.4%)	20 (14.9%)	15 (11.8%)	1.205 (0.554-2.621)	0.638
Implant	66 (25.3%)	29 (21.6%)	37 (29.1%)	0.708 (0.379-1.325)	0.28
Diaphragm/foam/jelly	16 (6.1%)	13 (9.7%)	3 (2.4%)	3.917 (1.051-14.6)	0.042
Condom	4 (1.5%)	1 (0.7%)	3 (2.4%)	0.301 (0.03-2.997)	0.306
Safe period	14 (5.4%)	6 (4.5%)	8 (6.3%)	0.678 (0.219-2.098)	0.5
Sterilisation	3 (1.1%)	2 (1.5%)	1 (0.8%)	1.808 (0.159-20.59)	0.633
None	99 (37.9%)	52 (38.8%)	47 (37%)	Ref	
Age of sexual debut					0.239
15-19	157 (60.2%)	84 (62.7%)	73 (57.5%)	1.569 (0.678-3.63)	0.293
20-29	65 (24.9%)	36 (26.9%)	29 (22.8%)	1.693 (0.675-4.243)	0.262
30-39	2 (0.8%)	1 (0.7%)	1 (0.8%)	1.364 (0.077-24.27)	0.833
<15	11 (4.2%)	2 (1.5%)	9 (7.1%)	0.303 (0.054-1.69)	0.173
Unknown	26 (10.0%)	11 (8.2%)	15 (11.8%)	Ref	
Leukocytes					0.013
++	9 (3.4%)	5 (3.7%)	4 (3.1%)	1.05 (0.275-4.011)	0.943
+++	22 (8.4%)	4 (3.0%)	18 (14.2%)	0.187 (0.061-0.569)	0.003
Negative	230 (88.1%)	125 (93.3%)	105 (82.7%)	Ref	

## Discussion

The findings from this study provide important insights into the epidemiology of HR-HPV in Kilifi County, particularly in the Rabai and Magarini sub-counties. The collection of genital swabs from a diverse group of women has resulted in a robust dataset for analyzing HPV prevalence and its associated risk factors in these regions. The age distribution of participants shows that a significant proportion (39.5%) belongs to the 30-39 age group, which is critical since this demographic is often at higher risk for cervical cancer due to cumulative HPV exposure. Additionally, the representation of younger women (15-29 years) highlights a key opportunity for early intervention strategies, including HPV vaccination and education on safe sexual practices. By focusing on specific sub-locations within Rabai and Magarini, the study emphasises the role of geographical factors in HPV transmission dynamics. Previous research has shown that environmental and socio-economic conditions can impact the prevalence of STIs, including HR-HPV. Identifying high-risk areas can help implement targeted public health interventions and allocate resources effectively. The high prevalence of HR-HPV calls for urgent improvements in cervical cancer screening programs and HPV vaccination initiatives in Kilifi County. Since HPV is a leading cause of cervical cancer, addressing this public health challenge through community-based education and accessible healthcare services is crucial. Furthermore, incorporating HPV testing into routine reproductive health services could enhance early detection and management of cervical pre-cancerous lesions.

Overall, the prevalence of HR-HPV was 48.7%, with a notable disparity between the Magarini and Rabai sub-counties. The Magarini sub-county exhibited a higher prevalence compared to the Rabai sub-county. The difference in prevalence between the wards in Rabai and Magarini sub-counties emphasizes the importance of implementing targeted public health interventions and improving screening programs to address and reduce the spread of HR-HPV. The varying prevalence across different wards and sub-locations within the sub-counties indicates that localised factors may contribute to the risk of HR-HPV infection. Such factors may include, but are not limited to, variations in sexual behaviour, cultural practices, health education, and access to healthcare services [26-28]. The prevalence of HPV within Kilifi County has been relatively understudied, despite unpublished reports indicating a high incidence of cervical cancer in the region. A pilot study conducted to evaluate the integration of HPV testing as a point-of-care service across nine counties in Kenya, including Kilifi County as one of the study sites, reported a HR-HPV prevalence of 34.7% at the Kilifi County Referral Hospital [20]. In comparison, our study found a higher prevalence of 48.7%. This could be attributed to our community-based sampling approach, which likely captured a more diverse demographic than the hospital-based screening. Moreover, the use of self-sampling resulted in higher participation rates among women in our study. Our findings also surpass the reported HR-HPV prevalence in sub-Saharan Africa of 26.5% from a systematic review of 17 studies published between 1999 and 2018 [29]. This highlights the importance of using community-based approaches to obtain a more accurate understanding of HPV prevalence.

Five out of the seven participants aged 15-19 years were HR-HPV positive, highlighting the importance of early vaccination and education on safe sexual practices. A higher prevalence was also observed in participants aged 20-29 years. This high prevalence in a relatively young age group suggests active sexual transmission and the need for targeted interventions, such as regular screening and awareness programs. Among the 261 participants, the largest cohort was in the 30-39 age group, with nearly half testing positive for HR-HPV. Although the prevalence is slightly lower compared to the younger age group (20-29 years), this may indicate a plateau effect attributed to increased immunity over time [30,31]. We also observed a high HPV prevalence among participants aged 40-49 years, suggesting a persistent risk of HPV infections in middle-aged women and the importance of routine screening and preventive measures. Even though the sample size for the 50-year-old group was small, with only six participants enrolled, three out of six tested positive, which suggests that HPV infection remains a concern even in older age groups. Our data demonstrates that HPV infection is prevalent across all age groups, with particularly high rates among younger women. This trend is consistent with other studies that show higher HPV prevalence in younger populations [30,32]. This study sheds light on the importance of assessing HPV prevalence across specific age groups to identify those at higher risk who require targeted interventions. Younger women are more likely to clear HR-HPV infections, while older women are at a higher risk of persistent infections, which can lead to cervical cancer [31]. Therefore, age-specific prevalence data helps us understand the risk across different age groups, aiding in the implementation of early detection and targeted prevention strategies.

Our study identified three primary HR-HPV strains: HPV 18, 45, and 16, along with a combined group of undifferentiated HR-HPV strains (HPV 26, 30, 31, 33, 34, 35, 39, 51, 52, 53, 56, 58, 59, 66, 67, 68, 69, 70, 73, 82, and 97). HPV types 16, 18, and 45 are considered high-risk due to their strong association with cervical and other anogenital cancers. These strains contribute significantly to the global burden of HPV-related diseases, making them a major public health concern. The observed prevalence of HPV 16, 18, and 45 is concerning as HPV 16 and 18 infections are attributed to 71% of invasive cervical carcinoma, and infection with HPV 16, 18, and 45 is attributed to 94% of cervical adenocarcinomas [4,7]. HPV 18 had the highest prevalence in this study. This strain is particularly linked to 37% of adenocarcinomas of the cervix, which are less common but more aggressive and harder to detect than squamous cell carcinomas. It is also linked to nearly 12% of cervical squamous cell carcinomas [33]. HPV 45 alone accounts for a notable percentage of cervical cancers, particularly adenocarcinomas. In our study, it was the second most prevalent HR-HPV strain. Although HPV 16 had the lowest prevalence in our study, it remains the most carcinogenic HPV type, attributed to 50% of cervical cancer cases worldwide [34]. The distribution of HPV strains differed between Rabai and Magarini sub-counties. In Rabai, HPV 16 was more prevalent, while HPV 18 and 45 were less common compared to Magarini. Other HR-HPV types were also less frequent in Rabai. On the other hand, the Magarini sub-county had a higher prevalence of HPV 18 and HPV 45. Additionally, Magarini had a greater number of other HR-HPV types. Co-infection rates were generally higher in Magarini, with significant numbers of co-infections involving HPV 18 and 45, as well as HPV 16, 18, and 45. These findings suggest variations in the patterns of HPV infection between the two sub-counties. This geographic variation can inform targeted public health strategies, vaccination campaigns, and screening programs tailored to the patterns observed in each subcounty.

Co-infections with multiple strains of HPV were found to be quite common, with the highest prevalence found in co-infection with HPV 18 and 45. This could be explained by HPV 45 being a member of the HPV 18-related alpha-7 species [35]. This co-infection of the two strains could potentially increase the risk of oncogene activation due to a higher viral load. The persistence of both strains is a crucial factor in cancer development. Additionally, the combined effect of these oncogenic strains may result in greater genomic instability within infected cells, increasing the likelihood of malignant transformation and accelerating the progression from pre-cancerous lesions to invasive cancer [36]. Similarly, the co-infection of HPV 16 and 18 indicates a significant overlap and potential synergistic effects that heighten the risk of cancer development, oncogenic activity, and genomic instability. Co-infection with HPV types 16, 18, and 45 was detected, and their combined activity may facilitate more aggressive cellular transformation, increasing the risk of high-grade lesions and cancer progression [36]. The presence of multiple high-risk types may necessitate more aggressive monitoring and treatment strategies. No co-infections were identified between HPV 16 and 45, suggesting distinct pathways or mechanisms of transmission and infection for these strains.

Chi-square analysis indicated that the type of sexual partnership (monogamous vs. polygamous) was significantly associated with HPV status. The OR indicates that individuals in monogamous relationships were less likely to test positive for HPV compared to those in polygamous relationships. This finding highlights the impact of sexual behavior on HPV transmission dynamics. Monogamous relationships may offer a protective effect due to a lower number of sexual partners, which reduces the risk of HPV exposure and infection. In contrast, polygamous relationships increase the number of sexual encounters, thereby increasing the risk of HPV transmission, as demonstrated in other studies [37,38]. There was also a significant association between leukocyte levels and HPV status, suggesting that higher leukocyte counts are correlated with HPV positivity in this study. Elevated leukocyte levels may indicate an immune response to infection, including HPV. Higher leukocyte counts in HPV-positive individuals could reflect an active immune response to combat the virus. This association underscores the potential role of immune system markers in understanding HPV infection and its progression. Elevated leukocyte levels in the cervical epithelium are known to increase the severity of HPV-induced lesions. This may, in turn, lead to inflammation, increased production of HPV oncogenes, and the release of pro-inflammatory cytokines [39]. Chronic inflammation may result in tissue injury and mutations in tumor suppressor genes, ultimately contributing to the development of HPV-induced cervical cancer [40]. Our findings suggest that leukocyte levels could serve as a biomarker for identifying HPV infection and progression to cervical cancer, aiding in early detection and management.

Our logistics regression model indicated that the use of diaphragm/foam/jelly as a contraceptive method is significantly associated with a higher likelihood of testing positive for HPV. The OR indicates that women who use these contraceptives are nearly four times more likely to be HPV-positive compared to those who do not use them. Although there is some uncertainty about the precise magnitude of the effect, as shown by the wide CI, the association remains statistically significant. This could be attributed to several factors, such as potential irritation or disruption of the vaginal mucosa, which might facilitate HPV infection or persistence. Moreover, the changes in the local vaginal microecology caused by these contraceptives can affect the vaginal flora, resulting in a reduction in the population of protective lactobacilli. This, in turn, makes the environment more vulnerable to infections, including HPV [41]. In comparison, a study conducted among female sex workers in Rwanda found an association between oral contraceptives and HPV infection [42]. Likewise, a study involving a cohort of 1,135 women in Thailand found that the use of combined oral contraceptives was significantly associated with HPV persistence [43]. A follow-up study conducted among 2,408 women with baseline HPV and cytological abnormalities found an association between injectable contraceptive use and HPV persistence [44]. This highlights the importance of considering contraceptive methods when evaluating HPV risk. The analysis also highlights the significant association between leukocyte levels and HPV status. Specifically, a strong correlation is observed between the presence of high leukocyte levels (+++) and HPV positivity. High leukocyte levels generally indicate an immune response or infection, and this finding could reflect the body's response to HPV or other infections that may influence susceptibility to HPV. However, no studies have reported high levels of leukocytes in HPV-positive women. Therefore, further investigation is needed to determine whether their presence in urine is significant.

Our study participants were drawn from a larger study investigating the effects of female genital schistosomiasis and HIV infection. This cross-sectional study targeted *S. haematobium* hotspots in Kilifi County, specifically the Rabai and Magarini sub-counties. Although the cross-sectional design was suitable for estimating prevalence, it limited causal interpretations. Consequently, the actual prevalence of HPV could be higher than reported. While self-sampling was well-received and encouraged greater participation, those who did not follow the instructions properly failed to provide quality samples for DNA extraction. Additionally, reliance on self-reported data may have introduced bias, especially concerning sensitive behavioral information. The localized sampling also limits the generalizability of findings to other populations. Further, the HPV testing kit used in this study was limited to detecting HPV types 16, 18, and 45, while all other subtypes were grouped as other HR-HPV types.

## Conclusions

In conclusion, our study indicates a high prevalence of HR-HPV in Rabai and Magarini sub-counties, Kilifi County, with varying prevalence in the different sub-counties. The reported HR-HPV genotypes also vary, suggesting potential differences in risk factors, health behaviours, and access to preventive measures. The combination of self-sampling and a community-based approach to HPV screening likely contributed to higher participation rates and increased diversity in our sample, capturing a broader demographic than traditional hospital-based screening. Therefore, this study highlights the importance of community-based approaches coupled with targeted screening programs within different age groups to improve the uptake of cervical cancer screening. The age-specific prevalence data from our study highlights the necessity for targeted interventions to mitigate the risk of cervical cancer. The high prevalence of HR-HPV types 18, 45, and 16 in our cohort further emphasizes the need for comprehensive genotyping in HPV testing to inform effective prevention and treatment strategies in the two sub-counties.
